# Characterization and Expression Analysis of Common Bean* Histone Deacetylase 6* during Development and Cold Stress Response

**DOI:** 10.1155/2017/2502691

**Published:** 2017-01-03

**Authors:** Rita Kusi-Appiah Hayford, Ayalew Ligaba-Osena, Mayavan Subramani, Adrianne Brown, Kalpalatha Melmaiee, Khwaja Hossain, Venu (Kal) Kalavacharla

**Affiliations:** ^1^Molecular Genetics and EpiGenomics Laboratory, Department of Agriculture and Natural Resources, Delaware State University, Dover, DE 19901, USA; ^2^Plant Biotechnology Laboratory, Department of Agriculture and Natural Resources, Delaware State University, Dover, DE 19901, USA; ^3^Center for Integrated Biological and Environmental Research (CIBER), Delaware State University, Dover, DE 1990, USA; ^4^Science and Mathematics Division, Mayville State University, Mayville, ND 58255, USA

## Abstract

Histone deacetylases (HDACs) are important regulators of gene transcription thus controlling multiple cellular processes. Despite its essential role in plants,* HDA6* is yet to be validated in common bean. In this study, we show that* HDA6* is involved in plant development and stress response. Differential expression of* HDA6* was determined in various tissues and the expression was seen to be upregulated with plant age (seedling < flowering < maturity). Higher expression was observed in flowers and pods than in stem, leaf, and root. Upregulation of* HDA6* gene during cold stress implies its prominent role in abiotic stress. Furthermore, the* HDA6* gene was isolated from three common bean genotypes and sequence analyses revealed homology with functionally characterized homologs in model species. The 53 kDa translated product was detected using an* HDA6* specific antibody and recombinant protein overexpressed in* Escherichia coli* showed HDAC activity* in vitro*. To our knowledge, this is the first report in the agriculturally important crop common bean describing the functional characterization and biological role of* HDA6*.

## 1. Introduction

In eukaryotes, epigenetic mechanisms regulate gene expression and play a significant role in many aspects of development [1]. The expression and activity of genes in eukaryotes are not only dependent on the genome but also dependent on the regulation conferred due to epigenetic marks such as DNA methylation and modification of histones. Histones are modified by the process of acetylation, phosphorylation, methylation, ubiquitination, ADP-ribosylation, glycosylation, or sumoylation [2] which subsequently affects interactions with DNA and thus gene expression. DNA methylation, histone modification, and chromatin remodeling regulate the accessibility of DNA to transcription factors and other regulatory proteins which affect transcriptional activities and gene expression. Chromatin is made up of DNA and histones as well as nonhistone proteins, which condenses DNA into the nucleus of a cell [3]. Approximately 147 base pairs of DNA wrap around an octamer of core histone proteins made up of two molecules each of H2A, H2B, H3, and H4. The N-terminal tails of histones provide the sites for histone modifications [4].

Chromatin in a closed state becomes inaccessible to transcription factors and other regulatory proteins. During acetylation, the positive charge of the lysine residues at the amino terminal tail is removed by addition of acetyl groups which reduces the electrostatic charge between the positively charged histone and negatively charged DNA molecule. In an open state, chromatin allows access of transcription factors to the promoter regions causing gene expression to occur, while the reverse occurs during deacetylation when transcription is inhibited resulting in a closed chromatin state [5]. Histone acetyltransferases (HATs) and deacetylases (HDACs) are, respectively, the enzymes that catalyze the addition and removal of acetyl groups from lysine residues on the histone N-terminal tails of histones [6]. Recent studies have revealed that some HDACs deacetylate nonhistone proteins besides histones [7]. HATs and HDACs are among the most well-characterized histone modification enzymes as compared to other histone modifiers and are grouped into distinct families. HDACs have been classified into three major distinct classes based on their homology to yeast HDACs, including Reduced Potassium Dependency-3/Histone Deacetylases 1 (RPD3/HD1), Silent Information Regulator 2 (SIR2), and the plant-specific Histone Deacetylase 2 (HD2). The* HDA6 *homolog in common bean which is the subject of this study belongs to the RPD3 superfamily and this family of HDACs has been widely studied among other families of HDACs. In* Arabidopsis*, the RPD3 family of HDACs apart from* HDA6* includes HDA7, HDA9, and HDA19.

A number of HATs and HDACs have been identified in the genome of various plant species including* Arabidopsis thaliana*, maize (*Zea mays*), rice (*Oryza sativa*), soybean (*Glycine max*), and barley (*Hordeum vulgare*) [8] and have been characterized. A total of 12 HATs and 18 HDACs have been identified in* Arabidopsis* [9]. In the rice genome seven HATs and 19 HDACs have been identified [10]. Likewise, 13 genes that encode HDACs have been identified in grapes (*Vitis vinifera*) and their expression analysis revealed specific functions during plant development [11]. Besides regulating chromatin structure, HDACs are also involved in plant growth and development [12]. Among the HDACs,* HDA6* is well studied and has also been shown to be involved in the control and maintenance of the genome, and the mechanisms and some targets of* HDA6* in gene silencing have been enumerated [13].

*HDA6* is involved in flower, seed, and leaf development in* Arabidopsis*. Mutants of* HDA6* in* Arabidopsis* showed late flowering time [14, 15]. In addition, reports show that* HDA6* is implicated in silencing transgenes and controls* Arabidopsis *ribosomal RNA [16]. There are reports on the involvement of the maize ZmRpd31 (RPD3-like enzyme, a member of the HDAC family) and ZmRBR1 (maize related protein) in regulating cell cycle progression, senescence, flowering, and suppression of embryonic features [17, 18]. Current reports also suggest that* HDA6* associates with small interfering RNAs from the RNA-directed DNA methylation pathway in gene silencing in* Arabidopsis* [19]. Additionally,* HDA6* together with methyltransferase 1 (MET1) are needed to silence centromeric regions in* Arabidopsis* [20].

HDACs are also known to respond to several biotic and abiotic stresses. For example, the* Arabidopsis* RPD3 group in the HDAC family has been implicated in stress signal response [21], while* HDA6* and HDA19 are involved in plant-pathogen interactions and associated with the jasmonic acid pathway [16]. A recent study from our group showed upregulation of common bean* HDA6* during inoculation with the bean rust pathogen,* Uromyces appendiculatus* race 53 [22].* HDA6* regulates chromatin in response to a number of environmental stresses. There are reports indicating the role of light detector Phytochrome B and* HDA6* in regulating light-controlled chromatin of Nuclear Organization Regions (NORs) [23].* HDA6* is also known to be involved in salt and cold tolerance in* Arabidopsis* [2, 24]. In relation to this work, cold temperatures cause considerable damage to plants and are one of the major abiotic stresses that affect the production of many crop plants including common bean [25].

Common bean is the most important food legume consumed worldwide; however biotic (62%) and abiotic (37–67%) factors affect the production of this crop causing significant yield losses [26]. The common bean genome has been sequenced and 473 Mb out of the ~587 Mb genomes have been assembled. It is a diploid with relatively large genome size compared to* Arabidopsis* (of ~135 Mbp) and serves as a model crop for other legumes. There are two major gene pools of common bean; Mesoamerican and Andean regions have distinct genetic and morphological characteristics [27, 28]. Given the availability of the genome sequence, it is important to understand the temporal and spatial expression as well as the physiological role of* HDA6*. Despite the involvement of HDACs in a myriad of biological processes, little is known about common bean HDACs. Therefore, we isolated* HDA6* from common bean and studied its temporal and spatial expression as well as its expression under cold stress. To our knowledge this is the first study characterizing the* HDA6 *gene and demonstrating differences in* HDA6* expression during development and abiotic stress (cold stress) in common bean.

## 2. Materials and Methods

### 2.1. Plants and Growth Conditions

*Phaseolus vulgaris* cultivar “Sierra” (Mesoamerican and resistant to* Uromyces appendiculatus* race 53), which was previously used for studying transcriptome and genome-wide histone binding analysis and global leaf HDAC activity [22, 29], was also used throughout the study.* Phaseolus vulgaris* genotypes “Olathe” (Mesoamerican and susceptible to* Uromyces appendiculatus* race 53) and “G19833” (Andean origin and reference genotype in Phytozome, http://www.phytozome.org) were used for cloning and sequence analysis. Seeds were germinated for five days on Petri dishes and irrigated with distilled water for another three days at room temperature. The seedlings were then transferred to poly pots filled with Promix soil. The plants were allowed to grow in the greenhouse under natural light until pod formation according to Ayyappan et al. 2015 [29], with samples collected as per the experimental protocol below.

### 2.2. Sample Collection for Spatial and Temporal Expression Analysis

Leaves were collected from the plants at seedling, flowering, and pod developmental stages when common bean plants were 2, 6, and 8 weeks old, respectively. Leaf tissues collected at the three different stages were used to study temporal expression of* HDA6.* Stems, roots, flowers, and pods were also collected for spatial expression analysis. Samples from three independent biological replicates were collected for each study. All tissues collected were flash frozen in liquid nitrogen and stored in −80°C for processing.

### 2.3. Cold Stress Treatment

Cold stress was imposed on two-week-old seedlings. One-half of the plants were treated with cold (4°C) while the other half was allowed to grow at an ambient growth condition (28/20°C) for 0, 24, and 72 h after which leaf tissues were collected. Samples from three independent biological replicates were collected for each treatment. All samples collected were immediately frozen in liquid nitrogen and subsequently stored in −80°C.

### 2.4. RNA Preparation and 1st-cDNA Synthesis

Total RNA was purified from various plant tissues and at different developmental stages using RNeasy Plant Mini Kit (Qiagen, Hilden, Germany) following the manufacturer's protocol. The concentration and purity of the RNA samples were determined using a Nanodrop spectrophotometer (Thermo Scientific, Wilmington, DE, USA). The integrity of all RNA samples was evaluated by gel electrophoresis. To eliminate contaminating genomic DNA, the RNA samples were treated with Deoxyribonuclease I (Invitrogen) following the manufacturer's instructions. The RNA samples were stored at −80°C until being used for downstream experiments. First-strand cDNA synthesis was performed using 10 *μ*g of DNase-treated total RNA using SuperScript III First-Strand Synthesis System (Invitrogen Life Technologies, Grand Island, NY, USA) following manufacturer instructions. The concentration of the cDNA samples was also checked spectrophotometrically.

### 2.5. Gene Cloning

The amino acid sequence of the* Arabidopsis HDA6* (*AtRPD3B*, Acc.* #* BAB10553) (Pandey et al. 2002) obtained from the National Center for Biotechnology Information (NCBI) was used as a query sequence to search homologs in the common bean (Andean genotype G19833) genome (http://www.phytozome.org) [30]. Fourteen hits were found in the genome of G19833 of which Acc. # BAB10553, which matched Phvul.003G203800.1, was the best hit. The sequence of* HDA6* was subsequently cloned from common bean and named* PvHDA6*. The coding region of* HDA6* was amplified from the cDNA using high fidelity Phusion Hot Start II DNA polymerase (Thermo Fisher Scientific, Inc., Grand Island, NY, USA) and gene-specific sense (Supplementary Table 1) primers designed by using DNASTAR software (DNASTAR, Madison, WI, USA). For amplifying common bean* HDA6,* 50 *μ*L PCR reaction contained 1 *μ*g of the cDNA, 400 nM of each forward and reverse primers, and one unit of the DNA polymerase. After initial denaturation at 98°C for 30 s, amplification was performed in 35 cycles of temperature gradient of 98°C for 30 s, 63°C for 30 s, and 72°C for 45 s and final extension for 10 minutes at 72°C and an infinite hold at 4°C. The PCR product was separated on agarose and purified using QIAquick gel extraction kit (Qiagen, Hilden, Germany) following the manufacturer's protocol. Since the polymerase produces blunt-end products, gel purified cDNA was cloned into the Zero Blunt® TOPO vector (Invitrogen Life Technologies, Grand Island, NY, USA) for subsequent sequencing.

### 2.6. Sequence Analysis

**Figure 1:**
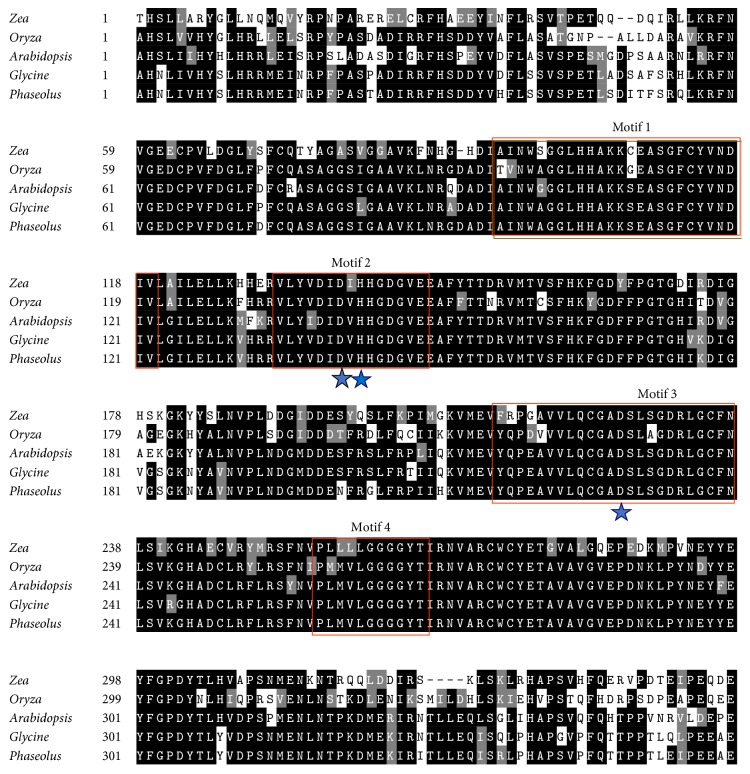
Multiple sequence alignment of putative* HDA6* from common bean cultivar Sierra with homologs in model plants. The alignment was generated using CLUSTALW and BOXSHADE and the amino acid sequences of* HDA6 *in* Phaseolus* used for the alignment are amino acids from Sierra. Highly conserved regions or identical amino acid regions from the BOXSHADE results are highlighted in black with white letters; gray regions are conserved amino acid substitutions. Four motifs were identified within the protein sequences (Pandey et al. 2002) and the three amino acids with star are conserved in common bean* HDA6* as in* Arabidopsis* HDA5 and they could be responsible for HDAC activity (Luo et al. 2015).

**Figure 2:**
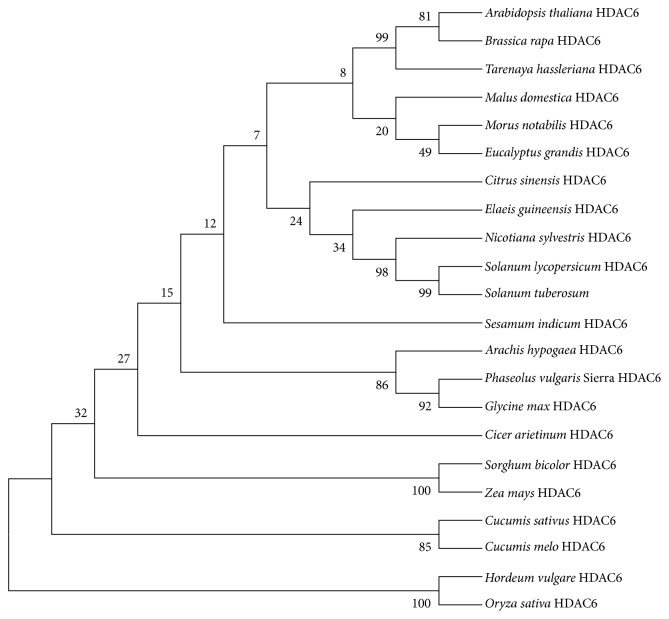
Phylogenetic analysis of* HDA6* in monocots and dicots. The neighbor-joining tree was constructed with mRNA sequences of plant* HDA6* using MEGA6. The tree indicates the evolutionary relationship between common bean* HDA6* and other dicot and monocot* HDA6. *The bootstrap analysis was deduced from 1000 replicates and Poisson correction method from MEGA version 6 software. The scientific names for the monocots and dicots used to construct the tree are as follows:* Glycine max (Gm), Arachis hypogaea (Ah), Cicer arietinum (Ca), Elaeis guineensis (Eg), Solanum lycopersicum (Sl), Sorghum bicolor (Sb), Hordeum vulgare (Hv), Sesamum indicum (Si), Oryza sativa (Os), Solanum tuberosum (St), Zea mays (Zm), Cucumis sativus (Cs), Morus notabilis (Mn), Tarenaya hassleriana (Th), Nicotiana sylvestris (Ns), Cucumis melo (Cm), Brassica rapa (Br), Citrus sinensis (Cs), Arabidopsis thaliana (At), *and* Phaseolus vulgaris* (Sierra). The monocots are considered as the outgroups for the phylogenetic analysis.

The nucleotide sequence of the cDNA was determined using Sanger sequencing method [31]. The sequences were compared with the reference sequence in Phytozome database (Phvul.003G203800.1.) using the SeqMan Pro module in DNASTAR Lasergene 10 Core Suite (DNASTAR, Madison, WI). The amino acid sequence was compared with the NCBI database using the Basic Local Alignment Search Tool (BLAST). Similarity of the common bean* HDA6* with homologs in other plant species was analyzed using online software CLUSTALW (European Molecular Biology Laboratory EMBL-EBI, Hinxton, Cambridgeshire, UK). Identity and similarity of amino acid residues were determined by BOXSHADE software (http://ch.embnet.org/software/BOX_form.html) with black shading representing identical amino acids while gray shading showing similar amino acids. The amino acid substitutions are found in the gray region as shown in Figure 1. The coding sequences of plant* HDA6* sequences in FASTA format were retrieved from the NCBI database. Prior to the phylogenetic analysis, the Molecular Evolutionary Genetic Analysis version 6.0 (MEGA6) was used to align the sequences based on conserved domains using CLUSTALW. The evolutionary history was inferred using the neighbor-joining method with 1000 bootstrap replicates by MEGA6 as shown in Figure 2 [32]. The evolutionary distance was computed using the Poisson correction method which is estimated by the number of amino acid substitutions per site. The coding data was translated assuming a standard genetic code table. All positions containing alignment gaps and missing data were eliminated only in pairwise sequence comparisons.

### 2.7. Quantitative PCR (qPCR)

First-strand cDNA was synthesized from DNase I-treated total RNA extracted from leaf tissues collected at seedling, flowering, and pod developmental stages as well as from plants subjected to cold stress. The concentration of the cDNA samples used for qPCR was normalized to 1 *μ*g/*μ*L. Quantitative PCR was performed to study the temporal and spatial expression of* HDA6 *using an ABI 7500 real-time PCR system and SYBR Green Kit (Applied Biosystems, Grand Island, USA). Twenty-five *μ*ls of the PCR reactions contained 1 *μ*g of 1st-strand cDNA, 2x Power SYBR Green Master Mix, and 0.15 *μ*L of* HDA6*-specific primers (Supplementary Table 2, in Supplementary Material available online at https://doi.org/10.1155/2017/2502691), and the constitutive* cons7* gene was used as an internal control. Relative expression was determined from three biological and three technical replicates using ΔΔC_T_ method (Applied Biosystems, Foster City, CA).

### 2.8. Recombinant* HDA6* Production in* E. coli*

For recombinant protein expression in* E. coli* the* HDA6* gene, which was previously subcloned into the Zero Blunt TOPO vector (above), was digested with* BamH1* and* Not1* restriction enzyme and ligated to a similarly digested pET24a plasmid (Novagen, Madison, USA), in frame with the C-terminus His-Tag coding sequence under the control of a strong bacteriophage T7 transcription and translation signals. The construct (*pET24a-HDA6) *and the control (empty vector control* pET24)* were then introduced into BL21 (DE3)* E. coli* competent cells (EMD-Millipore) suitable for high-level production of proteins from target genes cloned into pET vectors. Five milliliters of LB media containing 30 mg/L kanamycin was inoculated with a single colony of the BL21 (DE3) harboring* pET24-HDA6* or* pET24a *and allowed to grow overnight at 37°C. One milliliter of the overnight culture was used to inoculate 180 mL of LB containing 30 mg/L kanamycin. The cultures were allowed to grow until the optical density (OD_600_) reached 0.4–0.6. Protein expression was induced by adding 1 mM of isopropyl *β*-D-1-thiogalactopyranoside** (**IPTG) (Thermo Fisher Scientific, Grand Island, NY). Extraction of recombinant proteins was performed using the BugBuster protein extraction kit (Novagen, EMD-Millipore, Darmstadt, Germany) following recommended procedures. The concentration of the crude recombinant protein was determined by the Lowry method using Spectra Max® M5 reader (Molecular Devices LLC, VWR Corporate Headquarters, USA) [33].

### 2.9. Antibody Development and Western Blot

Total protein was isolated from leaves of plants at the developmental stages using trichloroacetic acid (TCA) acetone precipitation followed by SDS and phenol extraction as outlined by Wu et al. (2014) [34]. The protein concentration was measured using the Bradford method [35]. Equal amount of the proteins was loaded onto a SDS PAGE gel and transferred onto nitrocellulose membrane for western blot with the* HDA6*-specific antibody developed by GeneScript (Piscataway, NJ, USA) from the cloned* HDA6* (Hayford, Osena, and Kalavacharla 2014, unpublished). The antibody was diluted 1 : 1000 in TBST + 5% milk (http://www.bio-rad.com/webroot/web/pdf/lsr/literature/Bulletin_6376.pdf). meanwhile the secondary antibody-goat-anti-IgG (H+L) AP conjugate (Bio-Rad, USA) was diluted 1 : 5000 in TBST + 5% milk. The blot was developed using 25x AP Color developer (Bio-Rad Laboratories Inc., Hercules, CA, USA). The molecular weight of the protein was determined based on protein precision standard of known molecular weight.

### 2.10. Histone Deacetylase Enzyme Assays

To understand the real physiological role of common bean* HDA6*, the recombinant protein extracted from* E. coli* was purified using HIS-Select Affinity Gel (SIGMA-ALDRICH, St Louis, MO, USA) following the manufacturer's protocol.* In vitro* activity of the* HDA6* was determined using colorimetric assay following manufacturer's protocol (*BioVision* Inc., Milpitas, CA, USA) with slight modification and the assay was repeated three times.

### 2.11. Statistical Analyses

All experiments were repeated at least twice with three biological replicates. Statistical tests of significance of gene expression and deacetylase activity was performed using the PROC GLM procedure in SAS (Cary, NC, USA). Following significant *F*-tests, the Tukey multiple comparison procedure for all treatment pairs was used to separate the means [SAS] [36].

## 3. Results

### 3.1. Gene Cloning and Sequence Analysis

The* Arabidopsis* genome contains 12 HATs and 18 HDACs [9]. To find HDAC homologs in common bean, the amino acid sequence of the* Arabidopsis HDA6* (*AtPRD3B*, Acc. # BAB10553), obtained from the NCBI was used as a query sequence to search for homologs in the common bean genome at the Phytozome database (http://www.phytozome.org) using the BLAST tool. A total of 14 proteins belonging to the HDAC superfamily were found including Phvul.003G203800.1, which showed very high similarity to the* Arabidopsis HDA6* protein (*E*-value = 0.0). The nucleotide sequence of* PvHDA6* obtained from Phytozome was 1428 bp encoding a 475 amino acids' protein (Supplementary Figure 1). The gene was amplified from common bean cDNA synthesized from leaves of cultivar Sierra, Olathe, and G19833 followed by gel purification of the PCR product (Supplementary Figure 2) and cloning into Zero Blunt TOPO cloning vector for subsequent sequencing.

The* HDA6* homolog cloned from the cultivar Sierra and Olathe (both of Mesoamerican origin) contained six and three SNPs, respectively, as compared to the reference genome from cultivar G19833 of Andean origin (sequences are found in Supplementary Figure 4). However, these SNPs did not result in a change to the amino acid sequence. The amino acid sequence of the putative* HDA6* from both Sierra and Olathe are identical with the reference sequence from the cultivar G19833. A BLAST search of the cloned common bean* HDA6* sequences at NCBI returned the highest matches (99% identity with *e*-value = 0.0) with known* HDA6* genes, confirming that the gene cloned from common bean is indeed a homolog of the plant HDAC superfamily and was hence named* PvHDA6*. The amino acid sequences of Pv*HDA6* showed very high similarity with homologs in other plants such as* Glyma.05G040600.1 *(90.9%),* AtRPD3B or AtHDA6 *(77%), maize HD2 (74%), and rice HD2 (72%) (Figure 1). Furthermore, phylogenetic analysis established an evolutionary relationship between common bean and selected monocotyledonous and dicotyledonous* HDA6* genes.* PvHDA6* is very closely related to homologs in other legumes such as soybean, chickpea (*Cicer arietinum*), and peanut (*Arachis hypogea*) while it distantly related to that of cereals including rice, barley, sorghum (*Sorghum bicolor*), and maize (Supplementary Figure 4).

### 3.2. Expression Analysis of* HDA6* Gene at Different Developmental Stages

**Figure 3:**
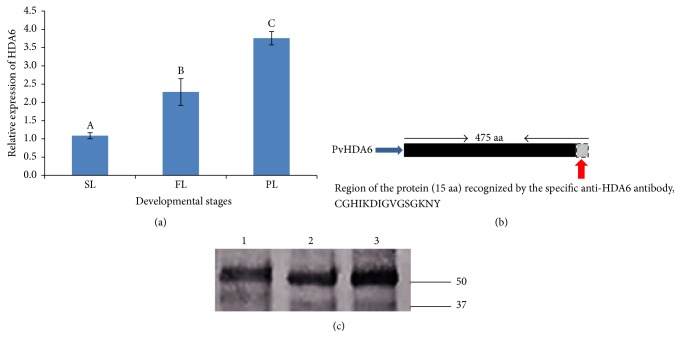
Validation of relative expression level of* HDA6* by qPCR using leaf tissues at different developmental stages of common bean. (a) Relative expression of* HDA6* gene in leaves at the seedling, flowering, and pod developmental stages of common bean. Three biological and three technical replicates were used for the analysis. The alphabets in the figure show that the expressions of* HDA6* in the leaf tissue at the different developmental stages are statistically significant at *p* < 0.05. (b) Diagram illustrating the common bean specific* HDA6* polyclonal antibody raised for protein validation. The 15 amino acids' peptide indicated in faded lines with red arrow is one of the three peptides selected for the epitope. (c) Western blot analysis using common bean specific* HDA6* antibody. The figure further validates the role of* HDA6* in common bean development.

To understand whether* PvHDA6* expression is affected by plant age, we studied the gene expression profile of this gene at different developmental stages (seedling, flowering, and pod) in the cultivar Sierra using qPCR. As shown in Figure 3(a) the* PvHDA6 *is constitutively expressed at all developmental stages. However, transcript accumulation increased significantly with age (*p* value < 0.0007).* PvHDA6 *is expressed higher at pod stage followed by flowering stage while expression at the seedling stage was lower compared to both pod and flower stages.

To confirm the expression at the protein level, we performed western blotting using anti-*HDA6* antibody on crude protein samples extracted from the leaf at seedling, flowering, and pod developmental stages. Expression at the protein level is consistent with that observed at the transcript level (Figure 3(b)). A translation product of 53 kDa was detected at all developmental stages with an increase in intensity in the order of seedling < flowering < pod stages.

### 3.3. Tissue-Specific Expression of* HDA6*

**Figure 4:**
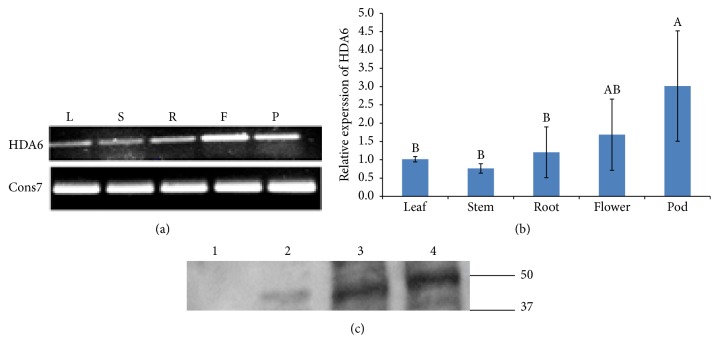
Spatial expression profile of* HDA6* in five tissues of common bean. (a) Traditional PCR was conducted using* HDA6* primers designed for qPCR. Each of the cDNA samples were normalized to 200 ng/*μ*L. Regular PCR was conducted using* HDA6* real-time primers designed for qPCR. The cons7 primer which is a constitutive gene was used as a control. (b) Determination of the relative expression of* HDA6* transcript in different tissues of common bean by qPCR. The alphabets indicate significant difference between common bean tissues at *p* < 0.05. (c) Western blot analysis using* HDA6* specific antibody with proteins isolated from empty pET vector (1) as a negative control, (2) leaf, (3) flower, and (4) pod.

To study the spatial expression of* PvHDA6*, RNA samples were isolated from various plant tissues (leaves, stems, roots, flowers, and pods) and used for synthesizing cDNAs, which were used for both traditional (Figure 4(a)) and quantitative PCR (Figure 4(b)).* PvHDA6* is constitutively expressed in all plant tissues. However, expression in the reproductive tissues (flower and pods) is higher than expression in other tissues. Expression in stem and the leaf is lower than the other tissues although the difference is statistically significant (*p* < 0.05) only for the pod.

### 3.4. *HDA6* Expression in Response to Cold

**Figure 5:**
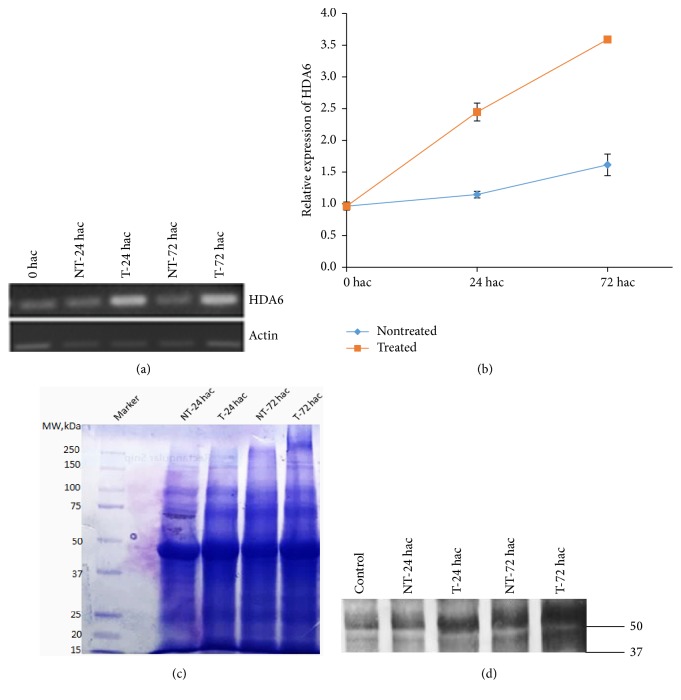
Expression analysis of* HDA6* after subjecting common bean plants to cold treatment at 4°C at different time points. Leaf tissues were collected from control and cold treated plants 24 and 72 hours after treatment. (a) PCR performed after RT-PCR of samples using* HDA6 *and* Actin* primers. (b) qPCR to determine relative expression of* HDA6* during cold stress. Three biological and three technical replicates were used for the analysis. Data were analyzed by analysis of variance (ANOVA) using [SAS] statistical software. Differences between treatments were assessed by Tukey's Studentized Range (HSD) Test for experiment. There was significant difference between control and cold treated samples at *p* < 0.0001. (c) Coomassie stained gel of proteins extracted from control and cold treated samples. (d) Western blot analysis using* HDA6* specific antibody with proteins isolated from control and cold treated samples.

**Figure 6:**
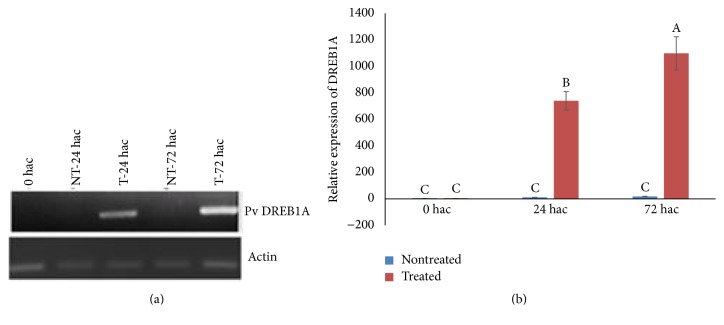
Expression analysis of DREB1A, a cold responsive gene after subjecting common bean plants to cold treatment at 4°C at different time points. Leaf tissues were collected from control and cold treated plants 24 and 72 hours after treatment. (a) cDNA PCR performed after RT-PCR of samples using DREB 1A and* Actin* primers. (b) qPCR to determine relative expression of DREB 1A during cold stress. Three biological and three technical replicates were used for the analysis. Data were analyzed by analysis of variance (ANOVA) using [SAS] statistical software. Differences between treatments were assessed by Tukey's Studentized Range (HSD) Test for experiment. The different letters A, B, and C indicate significant difference in gene expression between times of cold treatment at *p* < 0.0001. Values with the same letters are not statistically different.

Previous reports have shown that abiotic stresses such as drought, cold, and salt stress as well as phytohormones modulate expression of HDAC genes in various plant species [37, 38]. To understand the effect of cold stress on* PvHDA6* expression, two-week-old plants were subjected to cold stress treatment at 4°C, and gene expression was studied for 72 h. Interestingly,* PvHDA6* expression was significantly induced (*p* < 0.001) after 24 h of cold stress, while 72 h of cold stress further increased gene expression (Figure 5). To determine whether the plants are really experiencing cold stress condition, we also studied the expression of a DREB (dehydration-responsive element-binding proteins), which is a well-characterized transcription factor responsive to drought, salt, and cold stresses [35, 39]. Interestingly, expression of the DREB was also significantly (*p* < 0.001) induced by cold stress (Figure 6). Hence, increases in expression of* PvHDA6* may suggest that* HDA6* is a cold responsive gene.

### 3.5. Recombinant Protein Production and* In Vitro* Deacetylase Activity

**Figure 7:**
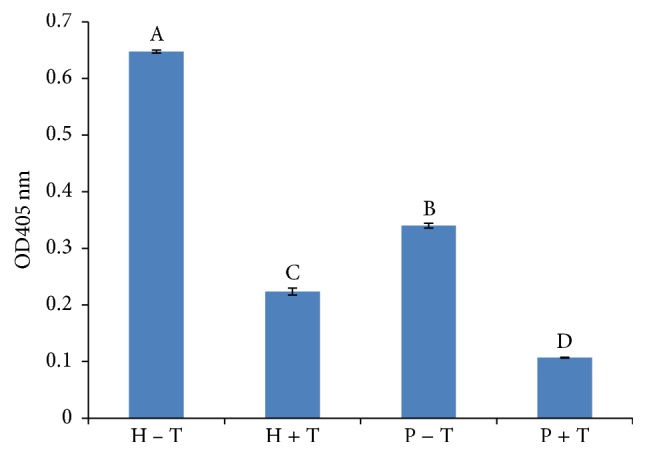
Histone deacetylase activity of recombinant* HDA6* protein from common bean. The* in vitro* activity was conducted with or without the addition of TSA (T) to purified recombinant* HDA6* protein (P) and positive control; HeLa nuclear extract (H). The alphabets indicate significant difference between protein and HeLa nuclear extract with TSA and without TSA (*p* < 0.05).

To deduce the physiological function of the common bean* HDA6*, the coding sequence was cloned into pET24a (+) vector for subsequent expression in* E. coli *(Supplementary Figure 3). The recombinant protein was extracted and purified using HIS-Select Ni-affinity gel and used for assaying deacetylase activity. The purified* HDA6* protein and a positive control HeLa nuclear extract, containing components of HDACs, were incubated with HDAC colorimetric substrate which contains an acetylated lysine side chain. The assay was carried out with or without trichostatin A (TSA), which is an organic compound that serves as an antifungal antibiotic and selectively inhibits class I and II mammalian histone deacetylase (HDAC) families of enzymes. As shown in Figure 7, the deacetylase activity of the recombinant Pv*HDA6* is lower than the positive control with or without TSA. Interestingly, deacetylase activity was proportionally inhibited in both the HeLa control and the* HDA6* proteins in the presence of TSA. This decrease in deacetylase activity in the presence of TSA is consistent with increased deacetylation, suggesting that the common bean* HDA6* is likely a functional member of the HDAC protein superfamily.

## 4. Discussion

Given that the HDACs are known to play a crucial role in plant growth and development as well as plant responses to environmental stimuli, we were interested in isolating and characterizing HDACs from common bean. In this study we report the isolation of* PvHDA6* and temporal and spatial expression of the gene as well as determination of its possible physiological function using recombinant protein produced in* E. coli*. This will widen our understanding of the regulatory mechanisms involved in growth and development of plants including responses to environmental stresses such as biotic and abiotic stresses.

### 4.1. Members of the* HDAC* Superfamily in Common Bean

In this study several members of the* HDAC* superfamily were identified in the common bean genome based on homology search using the* Arabidopsis HDA6*. HDAC proteins possess conserved histone deacetylase domains [9], suggesting that members of this family may be involved in histone deacetylation in plants. In common bean, nine distinct members of the HDAC superfamily have been identified (Supplementary Figure 5). Two of the sequences (PvSIR1 and PvSIR2) belong to the SIR2 family of NAD-dependent HDAC proteins, while one of the sequences (PvHD1) belongs to the plant-specific HD2 family. The remaining six proteins belong to the RPD3/HDA1 superfamily of HDACs which are further grouped into several classes (for review see Pandey et al. 2002 [9]). The presence of multiple proteins with HDAC conserved domains may suggest functional redundancy; alternatively, these proteins may have specific roles in various physiological processes or responses to environmental stimuli. However, the physiological function of each protein remains to be understood.

### 4.2. Temporal and Spatial Expression of* HDA6*

There is considerable evidence showing that HDACs regulate transcription and play significant roles in various plant developmental processes and response to environmental factors [40, 41]. To identify if the* HDA6* expression varies with plant age, we determined its expression profile at three developmental stages (seedling, flowering, and pod) using the quantitative PCR. Our findings revealed that the* HDA6* is constitutively expressed in all of the developmental stages. However, its expression was significantly induced (*p* < 0.001) with age (Figure 3). Gene expression was higher at the flowering stage as compared to the seedling stages. This level of expression was further increased as the plants reached maturity (pod stage). In line with the transcript data, the abundance of a ~53 kDa translation product increased with age. This suggests that* HDA6* may play a specific role at later developmental stages of the plants.

Previous reports have shown that expression of* HDA6 *in other plant species such as* Arabidopsis* is higher in reproductive tissues [3, 40]. Consistent with these reports, differential expression of* HDA6* in five tissues of common bean showed higher expression in the flowers and pod as shown in Figure 4. A tomato (*Solanum lycopersicum*) homolog of the* Arabidopsis HDA6* gene, SlHDA3, was more highly expressed in flowers though it was constitutively expressed in all stages of tomato development [4]. Enhanced expression of the common bean* HDA6 *during the reproductive phase as well as in reproductive tissues may suggest a possible role of* HDA6* in regulating flower initiation and development. Our suggestion is consistent with previous reports showing that downregulation of the* Arabidopsis AtRPD3B* (*HDA6*) delays flowering [42] while mutation of* HDA9* lead to early flowering [43], suggesting that the HDAC genes may function independently to regulate the flowering phenotype. The mechanism by which HDACs regulate flowering is not well understood. However,* Arabidopsis* HDACs have been shown to interact with various transcription factors to repress the expression of genes regulating developmental processes (for review see Zhao et al. 2016 [44]).* Arabidopsis HDA6* has also been shown to interact with* flowering locus D* to regulate flowering time [45]. Furthermore, a number of genes and transcription factors implicated in flowering time and reproductive transition have been identified in common bean including* Constans* (*CO*),* Early flowering 3* (*ELF3*),* flowering locus D*,* Gigantia* (*GI*), LEAFY (LFY), and* Terminal Flower* (*TFL*) [46]. Therefore, it remains to be understood whether* PvHDA6* modulates flowering time by activating or repressing expression of* flowering locus D* or other genes regulating the flowering traits. Furthermore, this study also revealed higher level of* HDA6* expression in the pods suggesting the possible role of* HDA6 *in pod or seed development [47].

### 4.3. Possible Involvement of* HDA6* in Cold Stress Response

Exposure of some plants to cold or low temperatures has been reported to induce epigenetic changes which may lead to changes in flowering time through vernalization [48]. The control of gene expression by HOS15 (WD4-repeat protein) through deacetylation associated with cold tolerance has been reported [49].* Arabidopsis HDA6* has been implicated in cold acclimation and freezing tolerance [24]. Likewise, our findings revealed upregulation of* PvHDA6* with prolonged exposure to cold temperature, suggesting that* PvHDA6* may be involved in cold responsive pathways as previously reported for* Arabidopsis HDA6* [24]. To substantiate this finding we studied the expression of DREB, which is a hallmark of stress-responsive gene expression [50]. We found that expression of DREB was also induced with cold stress (as shown in Figure 6). Upregulation of* HDA6* under cold stress may suggest its role in cold tolerance via direct regulation of tolerance genes or through interacting partners. For example, previous studies using the yeast two-hybrid system identified a number of interacting partners for HDA705 including RSS3, RHSF10, and GAMYB-binding protein, which are implicated in stress responses and hormone signaling in* Arabidopsis *[51].

### 4.4. Recombinant* HDA6* Possesses* HDA6* Activity

It has been reported that members of the RPD3/HDA1 family possess the deacetylase catalytic domain [4]. However, to date recombinant proteins of only a few members of RPD3/HDA1 including* Arabidopsis* HDA1 and HDA5 displayed varying degrees of histone deacetylase activity* in vitro* [52, 53]. Other members of the family including* HDA6*, HDA9, HDA15, and HDA17 (for review see [53]) did not show any HDAC activity which is suggested to be due to the absence of posttranslational protein modification machineries in the host* E. coli* or in the assay reaction [54].

In this study the recombinant Pv*HDA6* expressed in* E. coli* displayed HDAC enzymatic activity (Figure 7). Similar to HDA5 [53], enzymatic activity of the Pv*HDA6* was significantly inhibited by TSA. Luo et al. (2015) reported that site-directed mutagenesis revealed three critical amino acid residues (D198, D291, and H200) whose mutation abolished deacetylase activity of the recombinant HDA5. These three residues appear to be conserved among the HDAC proteins including Pv*HDA6*. The residues fall within motif 2 (D198 and H200) and motif 3 (D291) as reported in Pandey et al. (2002) [9]. It remains to be determined whether these residues are also critical for Pv*HDA6*. Taken together, our finding reveals that the common bean* HDA6* is a functional homolog of the HDAC superfamily with domains essential for deacetylase activity [9].

## 5. Conclusions 

We isolated* PvHDA6* from common bean which showed high similarity with HDACs in other plant species. Recombinant* HDA6* produced in* E. coli* displayed histone deacetylase activity. Future study will determine which of the conserved amino acid motifs are critical for its function using site-directed mutagenesis. Additionally, expression of the* PvHDA6* in common bean increases with age, predominately in reproductive tissues, and the expression is also induced by cold stress. Taken together,* PvHDA6* may be involved in regulating developmental processes and stress tolerance in common bean. However, further studies involving up- or downregulation of this gene in common bean may lead to deciphering the precise function and extent of regulation by* PvHDA6 in planta*. Furthermore, studies involving CHIP-seq, yeast two-hybrid or bimolecular fluorescent complementation would lead to identification of novel Pv*HDA6* interacting partners involved in key pathways in the regulation of common bean growth and development as well as biotic and abiotic stress tolerance.

## Supplementary Material

The supplementary file contains agarose gel pictures and coding sequences of amplification products cloning and sequence analysis of *HDA6* from common bean genotypes. Sequence alignment of* HDA6* sequence from *Arabidopsis* with predicted HDAC sequences in common bean obtained from phytozome database () revealed conserved domains. Lists of primers used for amplification and gene expression analysis of the studied genes are provided in the supplementary file.
