# Supplementary material for: Characterization and Expression Analysis of Common Bean Histone Deacetylase 6 during Development and Cold Stress Response
Source: Int J Genomics. 2017 Jan 3;2017:2502691. doi: 10.1155/2017/2502691 (PMC5239983; doi:10.1155/2017/2502691)
Supplement: Supplementary file 1 — The supplementary file contains agarose gel pictures and coding sequences of amplification products cloning and sequence analysis of HDA6 from common bean genotypes. Sequence alignment of HDA6 sequence from Arabidopsis with predicted HDAC sequences in common bean obtained from phytozome database () revealed conserved domains. Lists of primers used for amplification and gene expression analysis of the studied genes are provided in the supplementary file. [file 2502691.f1.dot]

Appendix A. Supplementary data


Characterization and expression analysis of common bean HISTONE DEACETYLASE6 during development and cold stress response
Rita Kusi-Appiah Hayford1, Ayalew Ligaba-Osena2, Mayavan Subramani1, Adrianne Brown1, Kalpalatha Melmaiee1,*, Khwaja Hossain3, Venu (Kal) Kalavacharlaa,*

1 Molecular Genetics and EpiGenomics Laboratory, College of Agriculture and Related Sciences, Delaware State University Dover, DE 19901, USA
2 Plant Biotechnology Laboratory, College of Agriculture and Related Sciences, Delaware State University Dover, DE 19901, USA
3 Science and Mathematics Division, Mayville State University, Mayville, ND 58255, USA
 *Center for Integrated and Biological Environmental Research (CIBER), Delaware State University Dover, DE 1990, USA
For correspondence: Venu (Kal) Kalavacharla, email: vkalavacharla@desu.edu 
Telephone: (302) 857-6492  
Fax: (302) 857-6402


Supplementary Figure 1. Amino acid sequence deduced from the nucleotide sequence of HDA6 from common bean cultivar Sierra


Supplementary Figure 2. 1% agarose gel of HDA6 using cDNA from Sierra- M: 1 kb ladder, lane 1: Sierra HDA6 from cDNA with amplicon size of 1428 bp and lane 2 is water to check for contamination. Amplification of the gene was done using a proof reading polymerase


Supplementary Figure 3. 1% agarose gels of sequential digest of insert (HDA6 from Sierra) before ligation into pET vector. A: shows 1% agarose gel of pET digested with first restriction enzyme which is BamH1 in NEB 4.0 buffer. B: Second digest of insert with Not1 using Cutsmart buffer. Inserts were first cloned in zero blunt TOPO vectors which are shown at the upper band and the lower band is the size of the gene. The band was excised, purified and ligated into pET vector which was also digested sequentially with BamH1 and Not1.  A linear band was obtained during the sequential digest of pET vector. Ligation products were introduced into DH5á competent cells and spread on LB plate with 50µg/µl of kanamycin. Bacterial colonies were then selected for colony PCR and the amplification result is shown in C. C: 1% gel of colony PCR showing positive colonies after ligation of insert in pET vector and transformation into DH5á. The lanes with no amplification represent false-positive colonies

Olathe_HDA6_ORF
ATGGGTATGATGGAAGAGGAGAGTAGCAATAGCAACATAGAAGGTGGGGCTTCGCTGCCATCGTCAGGTTCCGACGCCAAAAAGCGAAGAGTCACGTACTTTTACGAACCAACTATCGGCGATTACTACTACGGCCAGGGCCACCCAATGAAGCCGCACCGCATCCGCATGGCGCACAATCTTATCGTCCACTACTCCCTCCACCGCCGCATGGAGATTAACCGCCCTTTCCCGGCCTCCACCGCCGACATTCGCCGCTTTCACTCCGACGACTACGTCCACTTCCTCTCTTCCGTCTCCCCCGAGACCCTTTCGGACATCACCTTCTCGCGCCAACTCAAACGCTTCAACGTCGGCGAGGACTGCCCTGTCTTCGACGGCCTCTTCGACTTCTGCCAAGCTTCCGCTGGAGGGTCCATCGGCGCCGCCGTCAAACTCAACCGCGGTGACGCTGACATCGCCATCAATTGGGCCGGCGGCCTCCACCACGCCAAGAAGTCCGAAGCCTCTGGATTCTGTTACGTCAACGACATTGTTCTCGGTATCCTCGAGCTTCTCAAAGTTCACAGGCGAGTTCTGTATGTTGACATTGATGTTCACCATGGTGATGGAGTTGAGGAGGCCTTTTACACCACTGATAGAGTGATGACAGTCTCTTTTCACAAGTTTGGGGACTTTTTCCCTGGCACAGGGCATATCAAAGACATTGGGGTGGGCTCGGGAAAGAATTATGCGGTCAATGTCCCATTAAACGACGGAATGGATGATGAGAATTTTCGTGGTCTGTTTCGACCTATCATTCATAAAGTCATGGAGGTTTATCAACCTGAGGCAGTTGTTCTTCAATGTGGAGCTGATTCATTGTCTGGTGACAGGTTGGGTTGCTTCAACTTGTCTGTGAAAGGTCATGCAGATTGCCTTCGATTCCTTAGATCTTTCAATGTTCCTTTAATGGTTTTGGGTGGGGGTGGATATACAATTCGGAATGTTGCCCGTTGTTGGTGTTATGAGACAGCAGTGGCAGTAGGAGTGGAGCCTGACAATAAGTTGCCTTATAATGAATATTATGAATATTTTGGTCCAGATTATACTCTCTATGTCGATCCAAGCAACATGGAGAACCTAAACACACCCAAGGATATGGAAAAAATAAGGATCACACTACTAGAACAGATATCCCGTCTTCCCCATGCTCCCAGTGTACCTTTTCAGACAACACCACCTACCTTAGAAATTCCAGAAGAGGCTGAAGAGGACATGGATAGAAGACCAAAACTCCGCAAATGGGATGGTGAAGATTATGATTCTGACCCTGATGAAGGTGGAAAGGCTAATTCCAAGTTCTCAAATGTCAATGCCCATATGAGGGAAATTGTAGATGACATGGAAGAAGAGAAGCCAGGAGTGCATCCACCGTCTTGTTGTTGA

Sierra_HDAC6_ORF
ATGGGTATGATGGAAGAGGAGAGTAGCAATAGCAACATAGAAGGTGGGGCTTCGCTGCCATCGTCAGGTTCCGACGCCAAAAAGCGAAGAGTCACGTACTTTTACGAACCAACTATCGGCGATTACTACTACGGCCAGGGCCACCCAATGAAGCCGCACCGCATCCGCATGGCGCACAATCTTATCGTCCACTACTCCCTCCACCGCCGCATGGAGATTAACCGCCCTTTCCCGGCCTCCACCGCCGACATTCGCCGCTTTCACTCCGACGACTACGTCCACTTCCTCTCTTCCGTCTCCCCCGAGACCCTTTCGGACATCACCTTCTCGCGCCAACTCAAACGCTTCAACGTCGGCGAGGACTGCCCTGTCTTCGACGGCCTCTTCGACTTCTGCCAGGCTTCCGCTGGCGGGTCCATCGGCGCCGCCGTCAAACTTAACCGCGGTGACGCTGACATCGCCATCAATTGGGCCGGCGGCCTCCACCACGCCAAGAAGTCCGAAGCCTCTGGATTCTGTTACGTCAACGACATTGTTCTCGGTATCCTCGAGCTTCTCAAAGTTCACAGGCGAGTTCTGTATGTTGACATTGATGTTCACCATGGTGATGGAGTTGAGGAGGCCTTTTACACCACTGATAGAGTGATGACAGTCTCTTTTCACAAGTTTGGGGACTTTTTCCCTGGCACAGGGCATATCAAAGACATTGGGGTGGGCTCGGGAAAGAATTATGCGGTCAATGTCCCATTAAACGACGGAATGGATGATGAGAATTTTCGTGGTCTGTTTCGACCTATCATTCATAAAGTCATGGAGGTTTATCAACCTGAGGCAGTTGTTCTTCAATGTGGAGCTGATTCATTGTCTGGTGACAGGTTGGGTTGCTTCAACTTGTCTGTGAAAGGTCATGCAGATTGCCTTCGATTCCTTAGATCTTTCAATGTTCCTTTAATGGTTTTGGGTGGGGGTGGATATACAATTCGGAATGTTGCCCGTTGTTGGTGTTATGAGACAGCAGTGGCAGTAGGAGTGGAGCCTGACAATAAGTTGCCTTATAATGAATATTATGAATATTTTGGTCCAGATTATACTCTCTATGTCGATCCAAGCAACATGGAGAACCTAAACACACCCAAGGATATGGAAAAAATAAGGATCACACTACTAGAACAGATATCCCGTCTTCCCCATGCTCCCAGTGTACCTTTTCAGACAACACCACCTACCTTAGAAATTCCAGAAGAGGCTGAAGAGGACATGGATAGAAGACCAAAACTCCGCAAATGGGATGGTGAAGATTATGATTCTGACCCTGATGAAGGTGGAAAGGCTAATTCCAAGTTCTCAAATGTCAATGCCCATATGAGGGAAATTGTAGATGACATGGAAGAAGAGAAGCCAGGAGTGCATCCACCGTCTTGTTGTTGA

Supplementary Figure 4. The coding sequences of HDA6 after cloning from Sierra and Olathe


Phvul.006G057700.1    1 --MSLGYAEKLSYIEDVGNVGMVEYFDPSHVLREKIDQLAIMIKKSKHLVVFTGAGIS-T
Phvul.009G154300.3   58 VPRTFPRNEEKTFSNISRDKKTVPEADPPS--IKDVHLLYEFLDQSTKLTVLTGAGIS-T
Phvul.001G186300.1    1 ------------------------------------------------------------
Phvul.003G176700.1   157 SVANSST--AIGFDERMLLHAEVEKKSPPHPERPD--RLQ----AIA--ASLARAGIFPG
Phvul.003G185200.1   12 SSVNGQPRVGLLYDRRMCKHHT--PNNEDHVETPN--RIR----STW--NHLESAG-IPQ
Phvul.001G034500.2   72 YFDVSPSKVPLIYSESYDIAF--LGIEKLHPFDSS--KWG----RIC--RFLVSFGILDK
Phvul.008G283200.1    1 --MRSKDKIAYFYDGDVGSVY----FGAKHPMKPH--RLC----MTH--HLVLSYELHKK
Phvul.009G115300.1   12 -PDAVKRKVCYFYDPEVGNYY----YGQGHPMKPH--RIR----MTH--ALLAHYGLLQH
AtRPD3B              14 -PDGRKRRVSYFYEPTIGDYY----YGQGHPMKPH--RIR----MAH--SLIIHYHLHRR
Phvul.003G203800.1   24 -SDAKKRRVTYFYEPTIGDYY----YGQGHPMKPH--RIR----MAH--NLIVHYSLHRR


Phvul.006G057700.1   58 SCG-IPDFRGPKGIWTLQREGKA---------------------------------LPEA
Phvul.009G154300.3  115 ECG-IPDYRSPNGAYSSGFKPITHQEFLRSSRAR-----------RRYWA-----RSYAG
Phvul.001G186300.1    1 ------------------------------------------------------------
Phvul.003G176700.1  207 KCYSIPSREI-TPEELITVHSLEHIESVEVTTESLSSY------------FTPDTYANQH
Phvul.003G185200.1   61 RCLILEAKKA-EDKHLRLVHSRVHVNLIKNISSKQFSS-----RRPEIASKLNSIYFNEG
Phvul.001G034500.2  122 KCIVEP-LEA-SKDDLLVVHTELYLNRLKESSKVA-----MIVEVPPVAL-IPNCLVQQK
Phvul.008G283200.1   47 MEIYRP-HKA-YPVELAQFHSADYVEFLHRITPDTQHL--FLDELTKYNL-GEDCPVFDN
Phvul.009G115300.1   59 MQVHKP-FPA-RDRDLCRFHADDYVAFLRSITPETQQD--HLRQLKRFNV-GEDCPVFDG
AtRPD3B              61 LEISRP-SLA-DASDIGRFHSPEYVDFLASVSPESMGDPSAARNLRRFNV-GEDCPVFDG
Phvul.003G203800.1   71 MEINRP-FPA-STADIRRFHSDDYVHFLSSVSPETLSDITFSRQLKRFNV-GEDCPVFDG


Phvul.006G057700.1   84 SLPFHRAAPSLTHMALVELEKAGILKFVISQNV-DGLHLRSGIPREKLAELHGNSFMETC
Phvul.009G154300.3  158 WIRFTAAQPSAAHTALATLDKAGRIDFMITQNV-DRLHHRAGSN---PLEIHGTVYTVIC
Phvul.001G186300.1    1 ------------------------------------------------------------
Phvul.003G176700.1  254 SALAARLAAGLCAD-LASAIVSGRAKNGFALVR-PPGHHAGV-----------------R
Phvul.003G185200.1  115 SSEAAYLAAGSAVV-VVEKVASGELDSAVAIVR-PPGHHAEQ-----------------N
Phvul.001G034500.2  174 LLSPFRKQVGGTVL-AAKLA----KERGWAINVGGGFHHCSA-----------------E
Phvul.008G283200.1  102 IFEFCQIYAGGTID-AARRL--NNQLCDIAINWAGGLHHAKK-----------------C
Phvul.009G115300.1  114 LYSFCQTYAGGSVG-GAVKL--NHDQCDIAVNWAGGLHHAKK-----------------C
AtRPD3B             118 LFDFCRASAGGSIG-AAVKL--NRQDADIAINWGGGLHHAKK-----------------S
Phvul.003G203800.1  128 LFDFCQASAGGSIG-AAVKL--NRGDADIAINWAGGLHHAKK-----------------S

Phvul.006G057700.1  143 PSCGEEYFRDFEVETIGLKE------TS--------------------------------
Phvul.009G154300.3  214 IDCGYSFCRSLFQDQLKALN------PKWAEAIDNLDNGN--------------------
Phvul.001G186300.1    1 --------------------------------------------MEFW------------
Phvul.003G176700.1  295 QAMGFCLHNNAAVAALA----AQAAGAKRVLILDWDVHHGNGTQEIFEQNKSVLYISLHR
Phvul.003G185200.1  156 EAMGFCLFNNVAVAARYLLDERPDLGVKKILIVDWDVHHGNGTQKMFWNDSRVLFFSVHR
Phvul.001G034500.2  212 KGGGFCAYADISLCIHFAFV---RLNISRVMIIDLDAHQGNGHEMDFAYDSRVYILDMYN
Phvul.008G283200.1  142 EASGFCYINDLVLGILELL-----KHHPRVLYIDIDVHHGDGVEEAFYFTDRVMTVSFHK
Phvul.009G115300.1  154 EASGFCYVNDIVLAILELL-----KQHERVLYVDIDIHHGDGVEEAFYTTDRVMTVSFHK
AtRPD3B             158 EASGFCYVNDIVLGILELL-----KMFKRVLYIDIDVHHGDGVEEAFYTTDRVMTVSFHK
Phvul.003G203800.1  168 EASGFCYVNDIVLGILELL-----KVHRRVLYVDIDVHHGDGVEEAFYTTDRVMTVSFHK


Phvul.006G057700.1  165 ------------------------------------------------RRCTVAKCGTRL
Phvul.009G154300.3  248 -------PGSD----KSFGMKQR---------PDGDIEIDERFWEEDFIIPTCHKCNGVL
Phvul.001G186300.1    5 ------------------------------------------------------------
Phvul.003G176700.1  351 HEGGKFYPGTG--AAEEVGSMGAEGYCVNIPWSRGGVG-DNDYN--FAFQHVVLPIASEF
Phvul.003G185200.1  216 HEFGSFYPANDDGFYTKVGEGEGAGYNINVPWENGRCG-DADYF--AVWDHILLPVTKEF
Phvul.001G034500.2  269 -------PGI-----YPLDYEARNYINQKVEVKSG-TV-TEEYL--QKLDEALEVAGRRF
Phvul.008G283200.1  197 Y-GDQFFPGTG--DAKEIGEREGKFYAINVPLKDG-ID-DLSFT--RLFKTIISKVVETY
Phvul.009G115300.1  209 F-GD-YFPGTG--DVRDIGYGKGKYYSLNVPLDDG-ID-DESYH--FLFKPLIGKVMEVF
AtRPD3B             213 F-GD-FFPGTG--HIRDVGAEKGKYYALNVPLNDG-MD-DESFR--SLFRPLIQKVMEVY
Phvul.003G203800.1  223 F-GD-FFPGTG--HIKDIGVGSGKNYAVNVPLNDG-MD-DENFR--GLFRPIIHKVMEVY


Phvul.006G057700.1  177 KDTVLDWEDALPPKEMNPAEKHCKQADIVLCLGTSLQITP------ACNLPLKALRGGGK
Phvul.009G154300.3  288 KPDVVFFGDNVPKDRADMAMEASRRCDAFLVLGSSVMTMS------AFRLVRAAHEAGAA
Phvul.001G186300.1    5 ---------GVEVKSGQ-----SL------------------------------------
Phvul.003G176700.1  406 NPDFTIVSAGFDAARGD-----PLGCCDITPSGYAHMTHMLNGLSGG---KLLVILEGGY
Phvul.003G185200.1  273 NPDIIIVSAGFDAAVGD-----PLGGCRVTPFGYSVLLKELMNFAEG---RIVLILEGGY
Phvul.001G034500.2  313 NPELVIYNAGTDILEGD-----PLGRLEISPEGIALRDEKVFRFARERNIPIVMLTSGGY
Phvul.008G283200.1  250 QPGAIVLQCGADSLAGD-----RLGCFNLSIDGHS----ECVSFVKKFNLPLLVTGGGGY
Phvul.009G115300.1  261 RPGAVVLQCGADSLSGD-----RLGCFNLSIRGHA----ECVKYMRSFNVPLLLLGGGGY
AtRPD3B             265 QPEAVVLQCGADSLSGD-----RLGCFNLSVKGHA----DCLRFLRSYNVPLMVLGGGGY
Phvul.003G203800.1  275 QPEAVVLQCGADSLSGD-----RLGCFNLSVKGHA----DCLRFLRSFNVPLMVLGGGGY


Phvul.006G057700.1  231 V---------VIVNLQKTPKDKKASLVIHGFADKVIAGVMDHLNMQIPPFVRIDLFQIVL
Phvul.009G154300.3  342 T---------AIVNVGMTRADDFVPLKINARLGEILPRVLDMGSISIPAV*---------
Phvul.001G186300.1   15 ---KVDPGDDKIIHLSNAC----LGDVTKAKGGELVALNVKFGNQKLVLGTLS-------
Phvul.003G176700.1  458 NLRSISSSATA-------V----IKVLLGE-----------------SPGCEL-------
Phvul.003G185200.1  325 NLDSIARSMHA-------C----LEVLLKD-----------------KPVIRS-------
Phvul.001G034500.2  368 MKSSARVIADSIVNLSKKC----LIETSGA-----------------PKSS*--------
Phvul.008G283200.1  301 TKENVA-----------RC----WTVETGV-----------------LLDTEL-------
Phvul.009G115300.1  312 TIRNVA-----------RC----WCYETGV-----------------ALGIEV-------
AtRPD3B             316 TIRNVA-----------RC----WCYETAV-----------------AVGVEP-------
Phvul.003G203800.1  326 TIRNVA-----------RC----WCYETAV-----------------AVGVEP-------


Phvul.006G057700.1  282 VQALSNDKRYVNWTLQIASAHGQRAALPFIESVEVSFLDREDYKAAILDKQPFRLKRRTA
Phvul.009G154300.3      ------------------------------------------------------------
Phvul.001G186300.1   61 -----------------------SDKFPQISYDLIF---EKEFELS--------------
Phvul.003G176700.1  483 -----------------------ENSFPSKAGLV----------------------TVL-
Phvul.003G185200.1  350 -----------------------LEAYPFQSTWN----------------------VIQ-
Phvul.001G034500.2      ------------------------------------------------------------
Phvul.008G283200.1  322 -----------------------PNEIPENDYIKYF---APEFSLKIPNGQIENLNSKS-
Phvul.009G115300.1  333 -----------------------DDKMPEHEYYEYF---GPDYTLHVAPSNMENKNSRY-
AtRPD3B             337 -----------------------DNKLPYNEYFEYF---GPDYTLHVDPSPMENLNTPK-
Phvul.003G203800.1  347 -----------------------DNKLPYNEYYEYF---GPDYTLYVDPSNMENLNTPK-


Phvul.006G057700.1  342 YNKAFEMVLKLNFSDGCGCPSLEID-VPVDFK-VS--TDCFDFDKDYI-FQKLRDKAVLE
Phvul.009G154300.3      ------------------------------------------------------------
Phvul.001G186300.1   81 -------HSWKNGSVFFTGFKAQSQSESDN------DEDSDDFDEDIPVSAANG--KH-E
Phvul.003G176700.1  497 EVLKIQMKFWPSLGPIFVNL--ESQWR-----MYC--------------FEK--------
Phvul.003G185200.1  364 AVRQTLSPFWPTLASELPQE-LVSQIAPPPHTLISSSDSEDEDDKGAASLENVG--ELLE
Phvul.001G034500.2      ------------------------------------------------------------
Phvul.008G283200.1  355 YLSTIKMQVLENLRCIQHAPSVQMQEVPPDFY-IP-DFDEDEQNP---------------
Phvul.009G115300.1  366 LLEEIQSKLLENLSKLQHAPSVQFQERPPDSD-LG-EADEDH------------------
AtRPD3B             370 DMERIRNTLLEQLSGLIHAPSVQFQHTPPVNR-VL-DEPEDDMET---------------
Phvul.003G203800.1  380 DMEKIRITLLEQISRLPHAPSVPFQTTPPTLE-IP-EEAEEDMDR---------------

Supplementary Figure 5. Multiple sequence alignment of putative HDA6 from common bean (Phul.008G283200.1) and Arabidopsis HDA6 (AtRPD3B, Pandey et al., 2002) with genes belonging to the HDAC super families from common bean. The alignment was generated using ClustalW and BOXSHADE and the amino acid sequences of the HDACs in Phaseolus was obtained from Phytozome. Highly conserved regions or identical amino acid regions from the boxshade results are highlighted in black with white letters; gray regions are conserved amino acid substitutions


Supplementary Table 1. 
List of primers designed for amplification and sequencing of HDA6 common bean FL:  signifies primers for full length CDS of  genes, INT: internal primers used during sequencing of the genes, range of numbers: indicating regions in the sequences where primers were designed, nucleotides highlighted in green: extra nucleotides added to the 5 '  end of the restricion enzyme sites for efficient cutting of the primers and nucleotides highlighted in red: restriction enzymes sites added to the primers

HDA6-ORF-FL-BamHI /1-26- FW1: AAGGAAAGGAggattcATGGGTATGATGGAAGAGGAGAGTAG	
HDA6-ORF-INT/446-470-FW1: GTGACGCTGACATCGCCATCAATTG	
HDA6-ORF-INT/971-998-FW2: GATATACAATTCGGAATGTTGCCCGTTG	
HDA6-ORF-FL- NotI/1400-1425-RV1: TTCCTTCCTgcggccgcACAACAAGACGGTGGATGCACTCCTG	
HDA6-ORF-INT/339-366-RV1: GCAGTCCTCGCCGACGTTGAAGCGTTTG	
HDA6-ORF-INT/-1016-1044- RV2: ATTGTCAGGCTCCACTCCTACTGCCACTG	

Supplementary Table 2.s List of gene specific primers designed for qPCR using Gene Script Taqman software. Cons7 and Actin genes were used as the endogenous or control genes (Libault et al., 2008). DREB1B/CBF1 Arabidopsis sequences from To et al. (2011b) was used to find homologs in common bean. The primer pairs with asterisk were used in the expression analysis
Gene                          Forward 5ꞌ-3ꞌ                                  Reverse 5ꞌ-3ꞌ                                 	
	
Common bean HDA6   *CGCACAATCTTATCGTCCAC          *GACGTAGTCGTCGGAGTGAA         	
                         CCTGAGGCAGTTGTTCTTCA           TGCATGACCTTTCACAGACA         	
  Cons7              *atgaatgacggttcccatgta                  * ggcattaaggcagctcactct       
Common bean DREB  *GGAGGAGGAACTCAGACAAATG     *TCCTCAGCAACTCAAGCATATC
Actin                   *CAACCATGAGTGGAGTGACC         *TGGGAATGGAATCAAGTTCA                       	
